# Gene Expression Profiling of Mediators Associated with the Inflammatory Pathways in the Intestinal Tissue from Patients with Ulcerative Colitis

**DOI:** 10.1155/2020/9238970

**Published:** 2020-01-18

**Authors:** Gabriela Fonseca Camarillo, Emilio Iturriaga Goyon, Rafael Barreto Zuñiga, Lucero Adriana Salazar Salas, Ana Elena Peredo Escárcega, Jesús K. Yamamoto-Furusho

**Affiliations:** ^1^Inflammatory Bowel Disease Clinic, Department of Gastroenterology, Instituto Nacional de Ciencias Médicas y Nutrición Salvador Zubirán, México City, Mexico; ^2^MD/PhD (PECEM) Program, Facultad de Medicina, Universidad Nacional Autónoma de México, México City, Mexico; ^3^Department of Endoscopy, Instituto Nacional de Ciencias Médicas y Nutrición Salvador Zubirán, México City, Mexico

## Abstract

**Background:**

Multiple genes have been associated with IBD, and many of these can be linked to alterations in autophagy, UPR, ubiquitination, and metabolic and immune response pathways. The aim of this study was to analyze a transcriptomic panel of mediators associated with the inflammatory pathways in the colonic mucosa of UC patients. *Patients and Methods*. We studied a total of 100 patients with definitive diagnosis of UC (50 active and 50 in remission) and a control group (50 subjects) without endoscopic evidence of intestinal inflammation. Colonic mucosal biopsies were taken by colonoscopy and preserved in RNA later. Gene expression were measured by real-time polymerase chain reaction (RT-PCR).

**Results:**

The gene expressions of *XBP1*, *AGR2*, *HSPA5*, *UBE2L3*, *TNFRSF14*, *LAMP3*, *FCGR2A*, *LSP1*, *CTLA4*, *SOD2*, *TDO2*, and *ALDOB* mRNA levels were significantly higher in the colonic mucosa from UC patients (both quiescent and active) as compared to the control group (*P* < 0.05). Conversely, *IRGM*, *ORDML3*, *UBD*, *CUL2*, *CYLD*, *FOXC2*, *FOXO4*, *DOK3*, and *SNX20* mRNA levels were found to be significantly lower in patients with active disease, as compared to those with active disease (*P* < 0.05). Gene expressions of IRGM, CTLA4, FOXO4, SLC26A3, SLC39A4, SOD2, TDO2, and ALDOB were associated with clinical outcomes, such as medical treatment in response to aminosalicylates, histological remission, clinical course, and evolution.

**Conclusions:**

: The gene expressions of FOXO4, ALDOB, SOD2, TOD2, SLC26A3, and SLC39A4 were associated with the clinical course and histological activity and are of relevance since these provide the utility of new prognostic markers in IBD. Gene expression signature showed dysregulation in mediators associated with autophagy, ubiquitination, ER stress, oxidative stress, carbohydrate metabolism, solute transport, and T cell regulation in the colonic mucosa from patients with UC, suggesting that these genes could be involved in the pathogenesis of UC.

## 1. Introduction

Ulcerative colitis (UC) is caused by an aberrant immune response to environmental triggers in genetically susceptible individuals [1].

Recently, several new genes have been identified to be involved in the genetic susceptibility to inflammatory bowel disease (IBD) [2]. The gene expression characterization of novel molecules potentially will permit the identification of clinical assessment of phenotype therapeutic agents and prognosis in patients with IBD [2].

Dixon et al. suggest that important role of the analysis by novel gene signatures associated with clinical outcomes could help in better understanding of the molecular mechanisms involved in the IBD pathogenesis [3].

In 2012, genome-wide association studies (GWAS) have identified over 160 loci linked to increase IBD susceptibility. These loci implicate a diverse array of genes involved in IBD pathogenesis that encompass multiple physiological processes, including microbe recognition, lymphocyte activation, and intestinal epithelial defense [4].

Advances in discovery of new pathways involved in the etiopathogenesis of IBD highlight that the crucial role of the ubiquitination pathway-dependent autophagy targeting of intracellular pathogens has implications for pathogen growth and regulation of inflammatory responses by mediators of intestinal inflammation, T cell homeostasis, immune tolerance, epithelial barrier, and metabolic pathways.

The aim of the present study was to characterize transcriptomic panel of mediators associated with the inflammatory pathways such as autophagy, UPR, ubiquitination, metabolic and immune response in the colonic mucosa of UC patients to gain insight into the molecular pathways responsible for UC onset and progression.

## 2. Materials and Methods

### 2.1. Collection of Rectal Biopsies

Such as other previous studies, we collected relevant clinical and demographic information on all UC patients from medical records: gender, age at diagnosis, familial aggregation, smoking history, previous appendectomy, disease evolution, extension, extraintestinal manifestations, medical or surgical treatment, and clinical course of disease. All patients were included during the period from December 2012 to December 2015 belonging to the Inflammatory Bowel Disease Clinic at the Instituto Nacional de Ciencias Médicas y Nutrición Hospital [5].

The diagnosis of UC was done by the presence of the following criteria: a history of diarrhea or blood in stools and macroscopic appearance by endoscopy and biopsy compatible with UC [6–10].

We also included the control group consisted of noninflamed donors (no documentation of any disease) without evidence of endoscopic inflammation (mild chronic proctitis) and without histologically findings of inflammatory bowel disease. The control group did not take any medication. All individuals underwent colonoscopy for taking colonic biopsies before signing the written informed consent [6–10].

### 2.2. Sample Processing and Gene Expression Analysis

In order to isolate high-quality RNA, the rectal biopsy was taken by colonoscopy and immediately submerged in 0.5 ml of RNA later stabilization solution (Ambion, Austin, TX, USA) for storage and stored at -80°C until used. Then, total RNA was isolated using High Pure RNA Tissue (Roche Diagnostics, Mannheim, Germany) [6, 7].

For q-PCR assay quality control, determination of linearity and reproducibility was evaluated (VC < 10%). The mRNA relative quantification of target genes was conducted using the Light Cycler software 4.1, according to the 2ΔΔCt method.  Table 1 shows the details of the primer's designs and number of UPL (Universal Probe Library; Roche Diagnostics, Mannheim, Germany) used for the RT-PCR assay.

### 2.3. Ethical Considerations

The study was approved by the ethical committee of Instituto Nacional de Ciencias Médicas y Nutrición Salvador Zubirán (GAS-1485-15/18-1), and a written informed consent was obtained from all patients. This study was performed according to the principles expressed in the Declaration of Helsinki.

### 2.4. Statistical Analysis

Descriptive statistics were used as means and standard deviations. Kruskal-Wallis was used to test differences among groups and chi-squared test to assess the association between chemokine gene expression and clinical features. Odds ratio (OR) was used for evaluating the association. Statistical analysis was performed using the program SPSS ver. 19.

## 3. Results

### 3.1. Demographic and Clinical Characteristics

A total of 150 individuals were studied and divided in 3 groups: (1) active UC (*n* = 50); (2) remission UC (*n* = 50), and (3) control group without colonic inflammation (*n* = 50). The demographic and clinical characteristics of UC patients and controls are shown in Table 2.

### 3.2. Gene Expression Panels of Autophagy and Endoplasmic Reticulum Stress

**Figure 1:**
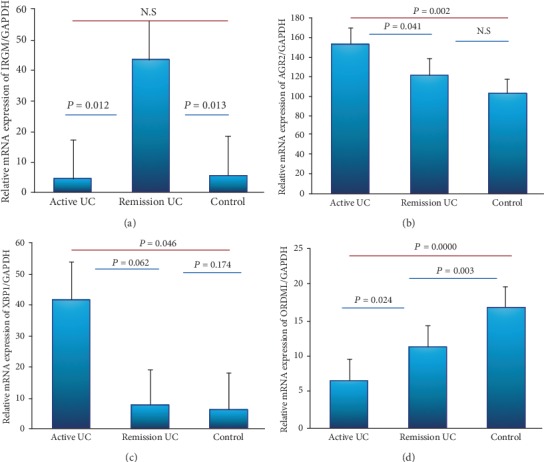
Gene expression profile of autophagy and endoplasmic reticulum stress in the colonic mucosa from patients with UC and controls. Gene expression. RT-qPCR was performed to assess mRNA levels in the colonic mucosa biopsies from UC patients; bars show means with standard error of (a) IRGM, (b) AGR2, (c) XBP1, and (d) ORDML3 transcript levels with GAPDH as housekeeping gene determined by 2*∆∆*Ct; differences among groups were assessed by Kruskall-Wallis test, and *P* values are presented in the figure. Statistical significance was considered when *P* value was <0.05.

The IRGM mRNA expression was detectable and quantifiable by RT-qPCR in colonic biopsies from UC patients and controls. These results showed that patients with UC in remission had significantly higher IRGM gene expression in mucosa compared to active patients and controls (*P* = 0.012 and *P* = 0.013, respectively). No differences were found between UC in remission and controls (Figure 1(a)). The medical treatment response to aminosalicylates (5-ASAs) was associated with high gene expression of IRGM (*P* = 0.001) as shown in Table 3.

Conversely, AGR2 expression was upregulated in the colonic mucosa from patients with active UC compared to UC patients in remission and controls (*P* < 0.04 and *P* < 0.002, respectively) as shown in Figure 1(b).

The XBP1 gene expression was increased in the colonic mucosa from patients with active and remission UC compared to the healthy control group (*P* = 0.062 and *P* = 0.046) Figure 1(c).

The ORDML3 expression was decreased in patients with active UC compared to UC patients in remission and the control group (*P* = 0.024 and *P* ≤ 0.001, respectively). The ORDML3 levels were decreased in UC remission compared to the control group of noninflamed donors (*P* = 0.003) as shown in Figure 1(d).

Interaction between ubiquitination pathway-dependent autophagy and inflammation in IBD has been proposed previously [9]; in addition, we decided to explore the gene expression of ubiquitin ligases in colonic tissue of patients with UC (Supplementary Figure 1).

### 3.3. Gene Expression Panel of Regulators of Intestinal Inflammation

**Figure 2:**
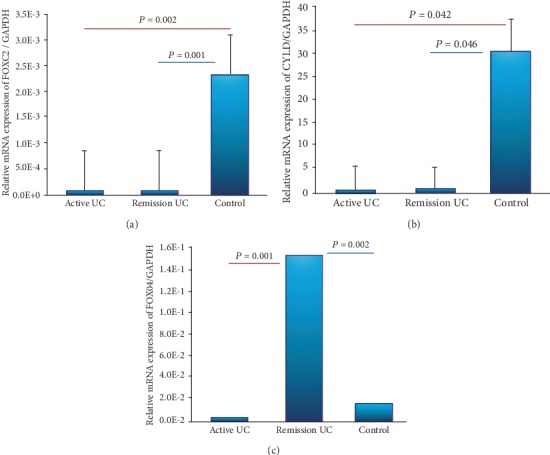
Gene expression panel of regulators of intestinal inflammation. Relative gene expression of were quantified by RT-PCR. (a) FOXC2, (b) CYLD, and (c) FOXO4 transcript levels with GAPDH as housekeeping gene.

The gene expression of FOXC2 was decreased in patients with UC in remission compared to normal controls without inflammation (*P* = 0.002) as well as in active UC compared to controls without inflammation (*P* = 0.001). No significant differences were found between UC patients in remission and active UC patients. The low expression of FOXC2 gene was not associated with any clinical outcome. The same form of CYLD gene expression was significantly decreased in active and remission UC groups compared to controls (*P* = 0.042 and *P* = 0.046) (Figures 2(a) and 2(b)).

The gene expression of FOXO4 was increased in patients with UC in remission compared to patients with active UC (*P* = 0.001) and controls (*P* = 0.002) as shown in Figure 2(c).The high expression of FOXO4 gene was associated significantly with histological remission in UC patients (*P* < 0.05, OR = 8.5, 95% CI: 0.83-87.8) as shown in Table 3.

### 3.4. Gene Expression Panel of T Cell Homeostasis and Peripheral Immune Tolerance

**Figure 3:**
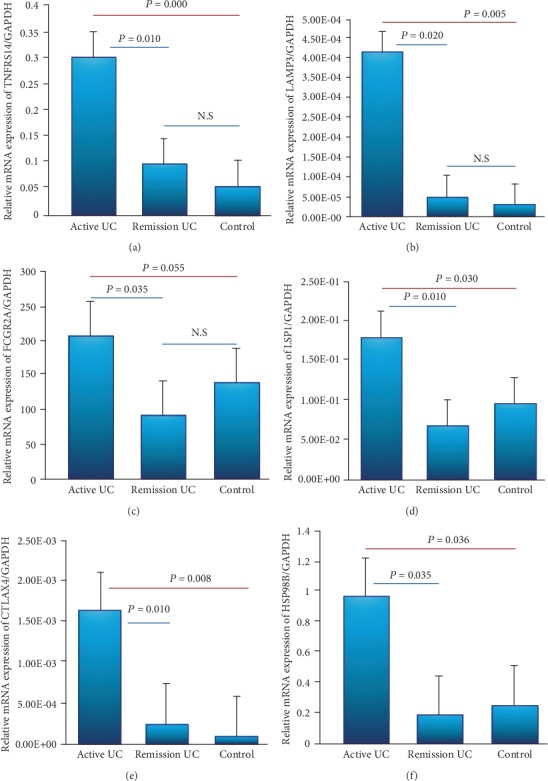
Gene expression panel of T cell homeostasis and peripheral immune tolerance in the colonic mucosa from patients with UC and controls. Relative gene expression was quantified by RT-PCR. Results were normalized using GADPH as housekeeping gene. (a) TNFRSF14, (b) LAMP3, (c) FCGR2A, (d) LSP1, (e) CTLA4, and (f) HSP90B transcript levels with GAPDH as housekeeping gene determined by 2*∆∆*Ct.

TNFRS14 gene expression was increased in patients with active UC compared with remission UC and normal controls (*P* = 0.01 and *P* ≤ 0.001). The LAMP3 expression was increased in patients with active UC compared to remission UC and control group (*P* = 0.020 and *P* = 0.0005). All patients with active UC had significantly higher FCGR2A gene expression in the colonic mucosa compared to remission UC patients and controls (*P* = 0.035 and *P* = 0.050, respectively) as shown in Figures 3(a)–3(c).

The gene expression of LSP1, CTLA4, and HSP90B1 was higher in patients with active UC compared to remission UC (*P* = 0.010, *P* = 0.008, and *P* = 0.036) and controls (*P* = 0.030, *P* = 0.010, and *P* = 0.035) as shown in Figures 3(d)–3(f). CTLA4 gene was associated with histological activity by Riley index score (*P* = 0.05, OR = 14, 95% CI: 0.83-235, Table 3).

Conversely, DOK3 and SNX20 gene expressions were decreased in active and remission UC groups compared to controls (*P* = 0.024 and *P* = 0.010) and (*P* ≤ 0.001 and *P* ≤ 0.001) (Supplementary Figure 2).

### 3.5. Gene Expression Panel of Solute Carrier Transporters

**Figure 4:**
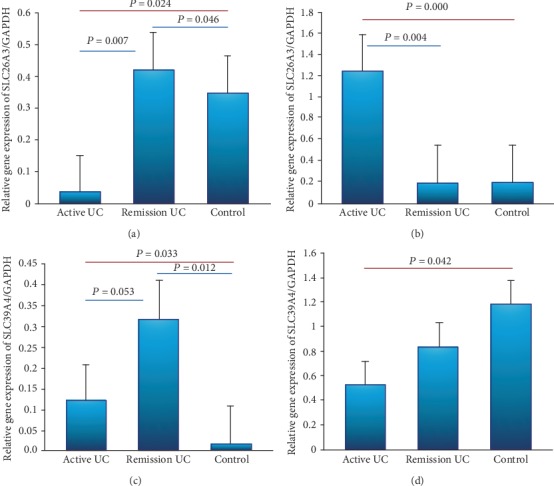
Gene expression panel of solute carrier transporters in colonic and ileal samples. Relative gene expression was quantified by RT-PCR. Results were normalized using GADPH as housekeeping gene. (a) SLC26A3 in rectal mucosal biopsy, (b) SLC26A3 in ileal mucosal biopsy, (c) SLC39A4 in rectal mucosal biopsy, and (b) SLC39A4 in ileal mucosal biopsy.

The gene expression of SLC26A3 was decreased in the colonic mucosa from patients with active UC compared with remission (*P* = 0.007) and control group (*P* = 0.024). Furthermore, decreased expression of the SLC26A3 gene was associated with a benign clinical course characterized by initial activity and then long-term remission (*P* = 0.001, OR = 0.05, 95% CI: 0.006-0.363, Table 3). Expression of SLC26A3 was increased in patients with UC remission compared to controls without inflammation (*P* = 0.046) as shown in Figure 4(a).

On the other hand, at the level of the terminal ileum, the expression of SLC26A3 was increased in patients with active UC compared with remission (*P* = 0.004) and controls (*P* ≤ 0.001) as shown in Figure 4(b).

Zinc is an essential nutrient that participates in various metabolic functions in the body such as the regulation of immune function and is a component of the inflammatory response. It is absorbed at the intestinal level and that is where zinc homeostasis occurs, where the Zip4 transporter responds and is encoded by the SLC39A4 gene [11].

The expression of SLC39A4 in the colonic mucosa was increased in patients with remission UC compared to control (*P* = 0.12). Furthermore, we detected an increased gene expression of SLC39A4 in patients with active UC compared with normal control group and remission UC (*P* = 0.0033 and *P* = 0.053, respectively) as shown in Figure 4(c). The elevated expression of the SLC39A4 gene was found to be associated with a long disease duration between 10 and 15 years (*P* = 0.0007, OR = .041, 95% CI: 0.005-0.299) as shown in Table 3.

In the terminal ileum region, gene expression of the SLC39A4 gene was significantly higher in the active group than in the normal control group without inflammation (*P* = 0.042) as shown in Figure 4(d).

The solute carrier family 11-member 1 gene (SLC11A1) is a divalent metal ion transporter with various pleiotropic effects on macrophage function. This gene that regulates iron, and is also regulated by cellular iron levels, has previously been linked to many infectious and autoimmune diseases [12].

The SLC11A1 gene expression was increased in the colonic mucosa from active UC patients compared with the remission UC and normal control groups (*P* = 0.014 and *P* = 0.03). No found differences in the SLC11A1 levels were decreased in UC remission compared to the control group shown in Supplementary Figure 3.

### 3.6. Gene Expression Panel of Oxidoreductase Enzymes

**Figure 5:**
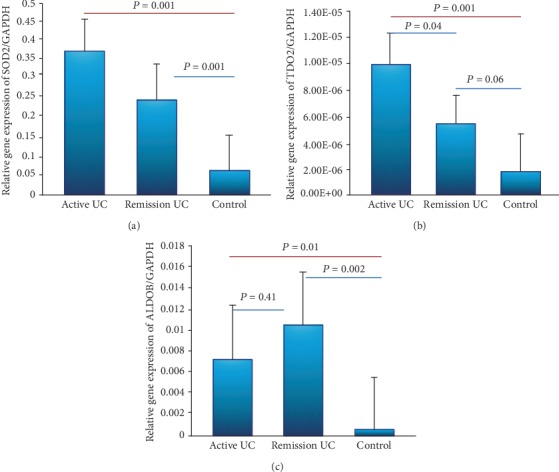
Gene expression panel of oxidoreductases enzymes. Relative gene expression was quantified by RT-PCR. (a) SOD2, (b) TDO2, and (c) ALDOB transcript levels with GAPDH as housekeeping gene determined by 2*∆∆*Ct; differences among groups were assessed by Kruskal-Wallis test, and *P* values are presented in the figure.

The SOD2 and TDO2 gene expressions were significantly higher in patients with active UC compared to the control group (*P* = 0.001 and *P* = 0.001) and remission UC and controls (*P* = 0.04 and *P* = 0.001) (Figures 5(a) and 5(b)). The gene expression of SOD2 was associated with severe histological activity (*P* = 0.00004, OR = 26.4, 95% CI: 4.4-157.2,) as shown in Table 3. The overexpression of TDO2 was associated with benign clinical course characterized by the presence of initial activity and then prolonged remission for more than 5 years (*P* = 0.06, OR = 4.7, 95% CI: 0.85-26, Table 3).

ALDOB gene expression was significantly higher in patients with remission UC compared with normal controls (*P* = 0.002). Furthermore, we also found significantly increased gene expression of ALDOB in active UC group compared with the control group (*P* = 0.01, Figure 5(c)).

Finally, ALDOB high gene expression was found to be associated with early age at diagnosis less than 40 years old (*P* = 0.005, OR = 1.6, 95% CI: 0.29-9.36) and benign clinical course characterized by the presence of initial activity and then long-term remission for at least 5 years (*P* = 0.005, OR = 0.52, 95% CI: 0.06-4.17, Table 3).

## 4. Discussion

The interest to study this panel of genes emerged from the great impact of the important role of different pathways such as endoplasmic reticulum stress, autophagy, ubiquitination, inflammation, and immunometabolism in the UC etiopathogenesis. This study analyzes gene expression profiling of mediators associated with the inflammatory response in the colonic mucosa from patients with UC.

The characterization of gene expression profiling of mediators associated with the inflammatory response potentially will provide the identification of new clinical assessment of phenotype in patients with IBD [1, 2].

In the present study, we evaluated epithelial expression of transcriptome of unfolded protein response (UPR) genes in the colonic mucosa from patients with UC. The expressions of XPB1 and AGR2 are enhanced in the inflamed mucosa of UC patients. Kasera et al. in 2010 discuss that genetic abnormalities within the components of the UPR genes that encode proteins reliant upon a robust secretory pathway (e.g., mucins) and environmental factors that create disturbances in the UPR are important factors in the primary development and/or perpetuation of intestinal inflammation [13].

Interestingly, the ORDML3 gene expression was decreased in patients with active UC compared to remission UC and normal control group as well as levels of ORDML3 were decreased in UC remission compared to the control group [6].

Conversely, patients with UC in remission had significantly higher IRGM gene expression in the colonic mucosa compared to active UC patients and normal controls. Fritz et al. [14] suggest that IRGM is required during the initiation phase of autophagy, when it localizes to bacteria-containing autophagy vacuoles. Basal levels of autophagy occur in essentially all cells as a homeostatic function in the process of protein and organelle turnover [14]. This increased expression of IRGM in patients with remission UC which suggests that this gene may be involved in controlling the inflammatory process and inducing mucosa healing in the autophagy process.

No significant differences were found in the mRNA levels of UPR genes (AGR2, XBP1, ORDML3, and IRGM) between active and remission UC groups. We should consider the fact that gene expression might be affected using anti-inflammatory drugs. It is possible to assert that the imbalance in AGR2 and XBP1 and the differential ORDML3 and IRGM expression in active and remission UC patients could be result of a clinical scenario such as remission or active disease.

The role of ubiquitin ligases and inflammation has been reported in patients with CD [15] but not in UC. In our study, we demonstrated an upregulation of gene expression of ubiquitin ligases such as HSPA5 and UBE2L3 in patients with active UC, Conversely, we found a downregulation of UBD and CUL2 genes in UC patients with colonic inflammation.

This could be explained by the mechanism described by Curtis et al. [16] that suggest an inflammatory response in diseases such as IBD is regulated by transcription factors (e.g., NF-*κ*B and HIF) that are posttranslationally modified to control the kinetics of their expression, primarily through proteasomal degradation pathways [15, 16]. Cullin 2 proteins, as components of ubiquitin E3 ligases, are neddylated for the polyubiquitination of effectors (e.g., I*κ*B and HIF-*α*). This neddylation reaction is regulated, in part, by the deneddylase DEN-1, as a mechanism to control E3 ligase activity [15].

This is the first depiction of the expression of UBD, UBE2L3, CUL2, and HSP5 genes in the colonic mucosa from patients with UC, suggesting that these genes could be involved in the pathogenesis of colonic inflammation in patients with UC.

Recent studies have shown that crosstalk between the innate and adaptive immune systems is crucial for this disease. However, recent advances in discovery of regulators of intestinal inflammation (FOXO4, FOXC2, and CYLD) and proinflammatory mediators highlight the crucial role of the adaptive immune response and T cell-mediated intestinal inflammation in inflammatory disorders.

Interestingly, we found a decreased gene expression of FOX proteins such FOXO4, FOXC2, and CYLD (regulators of intestinal inflammation) in patients with active UC compared to controls. The expression of FOXO4 was increased in patients with remission UC and was associated with histological remission. This result suggests the role of FOXO4 in the regulation of mucosal immunity in patients with UC.

Conversely, we reported an overexpression of TNFRSF14, LAMP3, FCGR2A, LSP1 and CTLA4, and HSP90B1 in the colonic mucosa from patients with active UC compared with remission and controls without inflammation.

Additionally, we found an overexpression of DOK3 and SNX20 genes in the colonic mucosa of patients with inactive UC compared with active UC and controls. A previous study showed the anti-inflammatory roles of Dok adaptors in chronic inflammatory diseases where DOK3 deficiency induces increase of TH2 cytokines in the asthma model mice [17]; our results suggest a potential anti-inflammatory role of DOK3 in the colonic mucosa in UC.

Additionally, we also explore the potential role of ion channels, transporters, and inflammatory response in patients with UC, and we evaluated the gene expression of solute carrier transporters in colonic and ileal samples. We found a decreased expression of SLC26A3 at the level of the colon and terminal ileum in patients with active UC.

Chatterjee et al. [18] demonstrated induction of the SLC26A3 gene expression in intestinal epithelial cells by CDX2. They showed that reduced expression of SLC26A3 in IBD-associated diarrhea may be, in part, be due to the downregulation of CDX2 in the inflamed colonic mucosa. Our results showed a downregulation of SLC26A3 gene expression in the colonic mucosa from patients with active UC and one study reported the altered transportome profile in patients with Crohn's disease [19]. This is the first description of SLC39A4 gene expression in patients with UC where SLC39A4 was increased in patients with remission UC compared to normal controls without inflammation; however, in the ileal tissue, this gene expression was increased in patients with UC. Furthermore, we also found elevated expression of the SLC39A4 gene, and it was associated with prolonged disease evolution between 10 and 15 years, which suggests the potential relation of *“*in situ*”* inflammation at the intestinal tissue and decreased function of the nutrients and mineral absorption suggesting that SLC39A4 could be used as nutritional therapeutic target in patients with active UC.

Conversely, we found an increased expression of SLC11A1 in the colonic mucosa from patients with active UC. SLC11A1 has been implicated in susceptibility to IBD, because it is a proton-coupled bivalent metal antiporter that is crucial in early macrophage activation [20]. Our results suggest an aberrant transcriptional regulation of solute carrier transporters in colonic and ileal samples from patients with UC and possible relation with a decreased absorption of the nutrients.

Micronutrient deficiencies such as zinc and folic acid are common among patients with IBD [21]. In this context, we explore the gene expression of micronutrient carrier FOLR1 and oxidoreductases enzymes (SOD2 and TOD2). No previous studies have evaluated the role of oxidoreductase enzymes' expression in the colonic mucosa from patients with UC.

The expression of the FOLR1 gene was significantly higher in the colonic mucosa of patients with remission UC compared to normal controls without inflammation and active UC.

These genes, TDO2 and SOD2, were upregulated in the colonic mucosa from patients with active UC. Even more, the gene TDO2 was associated with benign clinical course of UC and the gene expression of SOD2 was associated with histological activity in the colonic mucosa of UC patients. Finally, ALDOB gene expression is increased in patients with UC, and it was found to be associated with a benign clinical course and early diagnosis. This gene appears to be involved in the pathophysiology of UC which might contribute in carbohydrate metabolism during the inflammatory disease process.

This study showed the gene expression profiling of mediators associated with the inflammatory pathways in the colonic tissue from patients with active and remission UC.

It is important to consider transcriptomic profiles and their association with clinical outcomes for the possible application of this technology in the development of personalize medicine.

Several critical genes are involved in different pathways associated with the development and clinical outcomes in UC.

The genes expressions of FOXO4, ALDOB, SOD2, TOD2, SLC26A3, and SLC39A4 were associated with the clinical course and histological activity and are of relevance since these provide the utility of new prognostic markers in IBD. The association of these pathophysiological pathways opens the possibility of establishing new phenotypes in patients with IBD.

FOX04 gene expression was increased in patients with remission UC. FOX04 could be a potential marker of histological remission and its overexpression in remission UC might suggest an anti-inflammatory mechanism as an inhibitor of the production of NF-*κ*B.

Carbohydrate metabolism pathway by ALDOB gene expression was associated with early age at diagnosis of less than 40 years and prolonged benign clinical course characterized by the presence of higher initial activity and remission at 5 years.

Also, SOD2 and TDO2 gene expressions were associated with clinical course characterized by the presence of initial activity and then prolonged remission of more than 5 years.

Ion channels such the transporters SLC26A3 and SLC39A4 gene expressions were associated with clinical course and prolonged remission and evolution of disease.

These results can provide a new therapy targeting on the functions of these key genes and might provide novel perspective for IBD treatment.

The use of biomarker profiles could deliver clinically useful results in the next decade to help in personalizing care in patients with IBD. This could be a potential great benefit in predicting the course of disease in individual patients and in guiding appropriate medical therapy and stratifying the patient risk profile.

## Figures and Tables

**Table 1:** Oligonucleotide sequences for real-time RT-PCR.

Gene	Gene bank	Left	Right	UPL (Universal Probe Library)
AGR2	NM_006408.3	ggtgggtgaggaaatccag	gtaggagagggccacaagg	47
IRGM	NM_001145805.1	gcttgaaaaagagcagagcatt	gggcccaactgaagtgag	63
ORMDL3	NM_139280.1	tcaccaacctcattcacaaca	caaagggtgtccccttcac	4
XBP1^∗^	NM_005080.3, NM_001079539.1	ggagttaagacagcgcttgg	cactggcctcacttcattcc	37
HSPA5	NM_005347.4	aaaagcatttgggcagacc	ttaatggctgtattgggcttg	54
UBD	NM_006398.3	agagatggctcccaatgct	ggcatcaaaggtcattaaatcc	34
UBE2L3	NM_003347.2	atgcattctggggaaggag	ttgcggatttcttcaagctc	75
CUL2^∗^	NM_003591.3, NM_001198777.1, NM_001198778.1, NM_001198779.1	gaatgctgctccgagaaatc	ccccatggattactttctgg	22
FOXC2	NM_005251.2	ggggacctgaaccacctc	aacatctcccgcacgttg	3
CYLD^∗^	NM_001042412.1, NM_001042355.1, NM_015247.2	cagtctccggaatattctttgg	cagtgaaaccttgaccacga	11
FOXO4^∗^	NM_005938.3, NM_001170931.1	cgagggactggacttcaact	ggctcaagggtaaagagtagatatga	36
TNFRSF14	NM_003820.2	ctttgcctggacagctcct	cagcagagaggggctcag	3
LAMP3	NM_014398.3	tgctcatttttatgggattgc	tgagtttatttgatgccttcatctt	26
FCGR2A^∗^	NM_021642.3, NM_001136219.1	cctgtgaccatcactgtcca	agccacaatgatccccatt	44
LSP1^∗^	NM_001242932.1 NM_002339.2 NM_001013253.1 NM_001013254.1 NM_001013255.1	tccctaggcgtcccatct	ggcaacaggaagcaacttct	81
CTLA4^∗^	NM_005214.4 NM_001037631.2	ttcatccctgtcttctgcaa	agtggctttgcctggagat	40
HSP90B1	NM_003299.1	tcctatttatgtatggagcagcaa	gcagcttcatcatcagattcttc	69
DOK3^∗^	NM_024872.2 NM_001144875.1 NM_001144876.1	tccccatggaggaaaactc	caccaccacgggaaactc	63
SNX20^∗^	NM_001144972.1 NM_153337.2	cacacccggactgatgtgt	tctctgttagtggtgcctgga	76
SLC26A3	NM_000111.2	ccatcatcgtgctgattgtc	agctgccaggacggactt	2
SLC39A4	NM_017767.2 NM_130849.2	gctccagtgtgtgggaca	gcctgttccgacagtcca	46
FOLR1^∗^	NM_016729.2 NM_000802.3 NM_016725.2 NM_016724.2	gaggacaagttgcatgagca	cctggctggtgttggtaga	65
SOD2^∗^	NM_001024465.1 NM_001024466.1	aatcaggatccactgcaagg	taagcgtgctcccacacat	3
TDO2	NM_005651.2	ggagaagaaaatgaactgctacttaaa	ggctctaaacctggagttctttc	15
ALDOB	NM_000035.3	cggccaaaggacagtatgtt	aagagcgactgggtggaa	45
GAPDH	NM_002046.3	agccacatcgctcagacac	gcccaatacgaccaaatcc	60

Assays were designed to detect both transcript isoforms. UPL: Universal Probe Library.

**Table 2:** Clinical and demographic characteristics of ulcerative colitis patients and controls.

Patients number, gender (F/M)	(52/48)
Age (years range)	(20-75)
Disease duration (1-3/>3 years)	(39/61)
Disease activity (active/remission)	(50/50)
Disease extension (Montreal classification)	
E1-proctitis/E2-left-sided colitis/E3-pancolitis	(35/9/56)
Endoscopic activity (inactive/mild/moderate/severe)	(50/26/12/12)
Histological activity (inactive/mild/moderate/severe)	(50/24/14/12)
Current therapy: 5-aminosalicylate/corticosteroids	(69/31)
Extraintestinal manifestations (without/arthritis/others)	(60/24/16)
Control group		
Number of patients, sex (F/M)	(21/39)
Age (year range)	(19-70)
Treatment (5-aminosalicylate/corticosteroids)	(0/0)

**Table 3:** Gene expression associates with clinical outcomes.

Gene expression	*P* value	Association with clinical outcome	Pathways involved with IBD
↑ IRGM	*P* = 0.001	Medical treatment response to aminosalicylates (5-ASAs)	Autophagy
↑ FOXO4	*P* = 0.05	Histological remission	Immune response
↑ CTLA4	*P* = 0.05	Histological activity index score	
↓ SLC26A3	*P* = 0.001	Clinical course: the course of the same initial activity and prolonged remission	Solute transport
↑ SLC39A4	*P* = 0.0007	Evolution: tendency to years of evolution of between 10 and 15 years	
↑ SOD2	*P* = 0.00004	Severe histological activity	Oxidative stress
↑ TDO2	*P* = 0.06	Clinical course: characterized by the presence of initial activity and then prolonged remission more than 5 years	
↑ *ALDOB*	*P* = 0.005	Evolution: early age at diagnosis of less than 40 years	Carbohydrate metabolism
↑ *ALDOB*	*P* = 0.005	Clinical course: prolonged benign clinical course characterized by the presence of higher initial activity and remission at 5 years	

## Data Availability

All data and figures used to support the findings of this study are included within the article.

## Abbreviations

AGR2: Anterior gradient 2, protein disulphide isomerase family member
ALDOB: Aldolase, fructose-bisphosphate B; CD, Crohn's disease
cDNA: Complementary DNA
CTLA4: Cytotoxic T-lymphocyte associated protein 4
CUL2: Cullin 2
dNTPs: Deoxyribonucleotide triphosphate
FCGR2A: Fc fragment of IgG receptor IIa
FOLR1: Folate receptor 1
FOXC2: Forkhead box C2
FOXO4: Forkhead box O4
GAPDH: Glyceraldehyde-3-phosphate dehydrogenase
GWAS: Genome-wide association studies
HSP90B1: Heat shock protein 90 beta family member 1
HSPA5: Heat shock protein family a (Hsp70) member 5
IBD: Inflammatory bowel disease
IRGM: Immunity related GTPase M
LAMP3: Lysosomal associated membrane protein 3
LSP1: Lymphocyte specific protein 1
ORMDL3: ORMDL sphingolipid biosynthesis regulator 3
RT-PCR: Real time polymerase chain reaction
SLC26A3: Solute carrier family 26 member 3
SLC39A4: Solute carrier family 39 member 4
SNX20: Sorting nexin 20
SOD2: Superoxide dismutase 2
TDO2: Tryptophan 2,3-dioxygenase
TNFRSF14: TNF receptor superfamily member 14
UBD: Ubiquitin D
UBE2L3: Ubiquitin conjugating enzyme E2 L3
UC: Ulcerative colitis
XBP1: X-box-binding protein 1.

## Supplementary Materials

Supplementary Materials Supplementary Figure 1: gene expression panel of ubiquitin ligases in colonic mucosa. Results were normalized using GADPH as housekeeping gene. Supplementary Figure 2: gene expression panel of peripheral immune tolerance. (a) SNX20 and (b) DOK3 transcript levels. Supplementary Figure 3: gene expression of SLC11A1 in the colonic mucosa. Results were normalized using GADPH as housekeeping gene. Transcript levels with GAPDH as housekeeping gene determined by 2*∆∆Ct*.
