# Supplementary material for: Gene Expression Profiling of Mediators Associated with the Inflammatory Pathways in the Intestinal Tissue from Patients with Ulcerative Colitis
Source: Mediators Inflamm. 2020 Jan 18;2020:9238970. doi: 10.1155/2020/9238970 (PMC7201440; doi:10.1155/2020/9238970)
Supplement: Supplementary Materials — Supplementary Figure 1: gene expression panel of ubiquitin ligases in colonic mucosa. Results were normalized using GADPH as housekeeping gene. Supplementary Figure 2: gene expression panel of peripheral immune tolerance. (a) SNX20 and (b) DOK3 transcript levels. Supplementary Figure 3: gene expression of SLC11A1 in the colonic mucosa. Results were normalized using GADPH as housekeeping gene. Transcript levels with GAPDH as housekeeping gene determined by 2∆∆Ct. [file 9238970.f1.pdf]

Supplementary figures

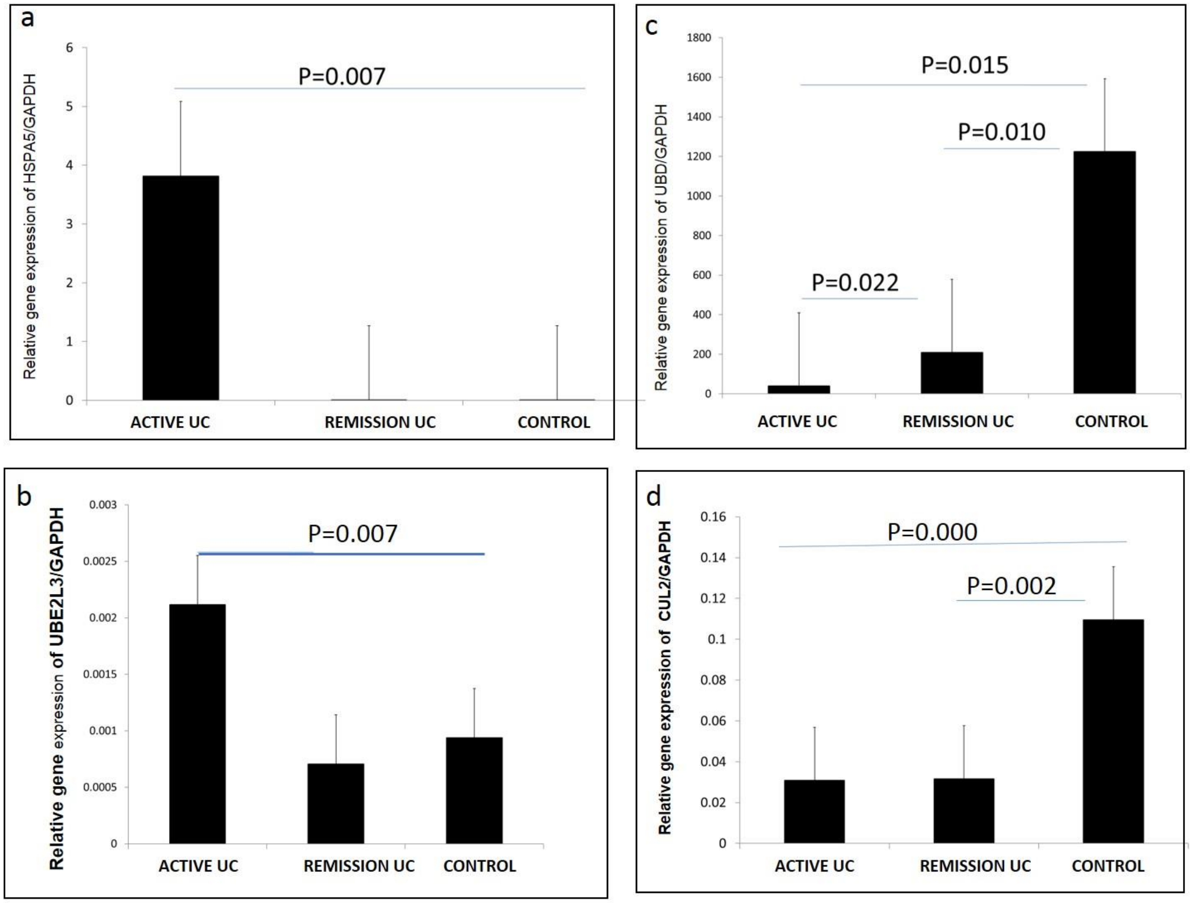

Figure 1s. Gene expression panel of ubiquitin ligases in colonic mucosa. Results were normalized using GADPH as housekeeping gene. a) HSPA5, b) UBE2L3, c) UBD, d) CUL2 transcript levels and p values are presented in the figure.

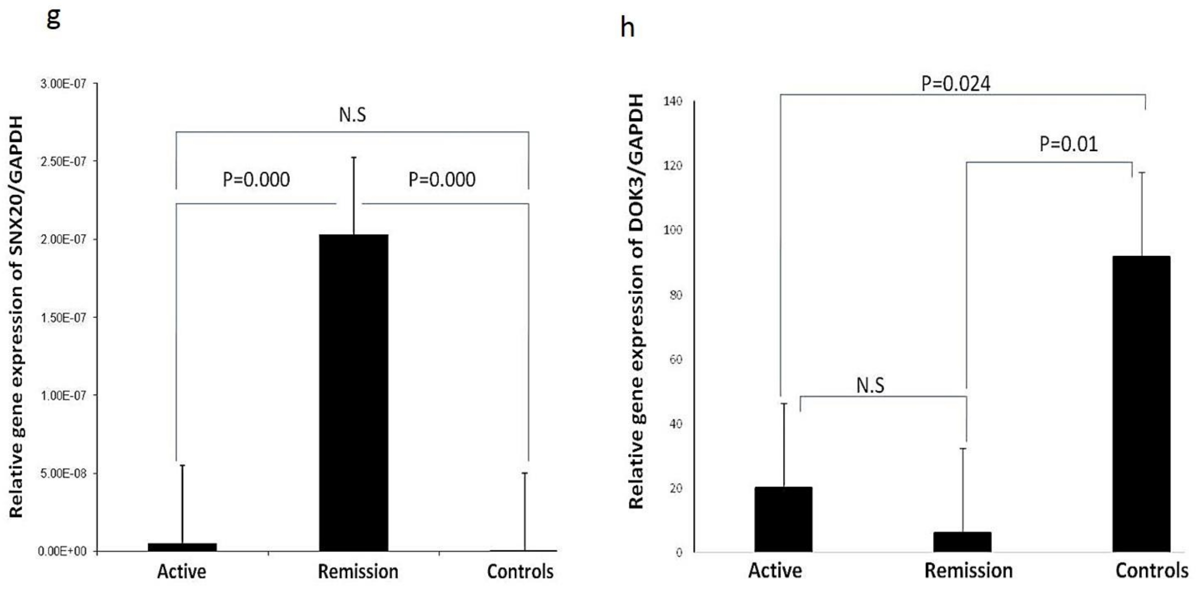

Figure 2s. Gene expression panel of peripheral immune tolerance. a) SNX20 and b) DOK3 transcript levels with GAPDH as housekeeping gene determined by 2 Δ ΔCt, differences among groups were assessed by Kruskall Wallis test, and p values are presented in the figure.

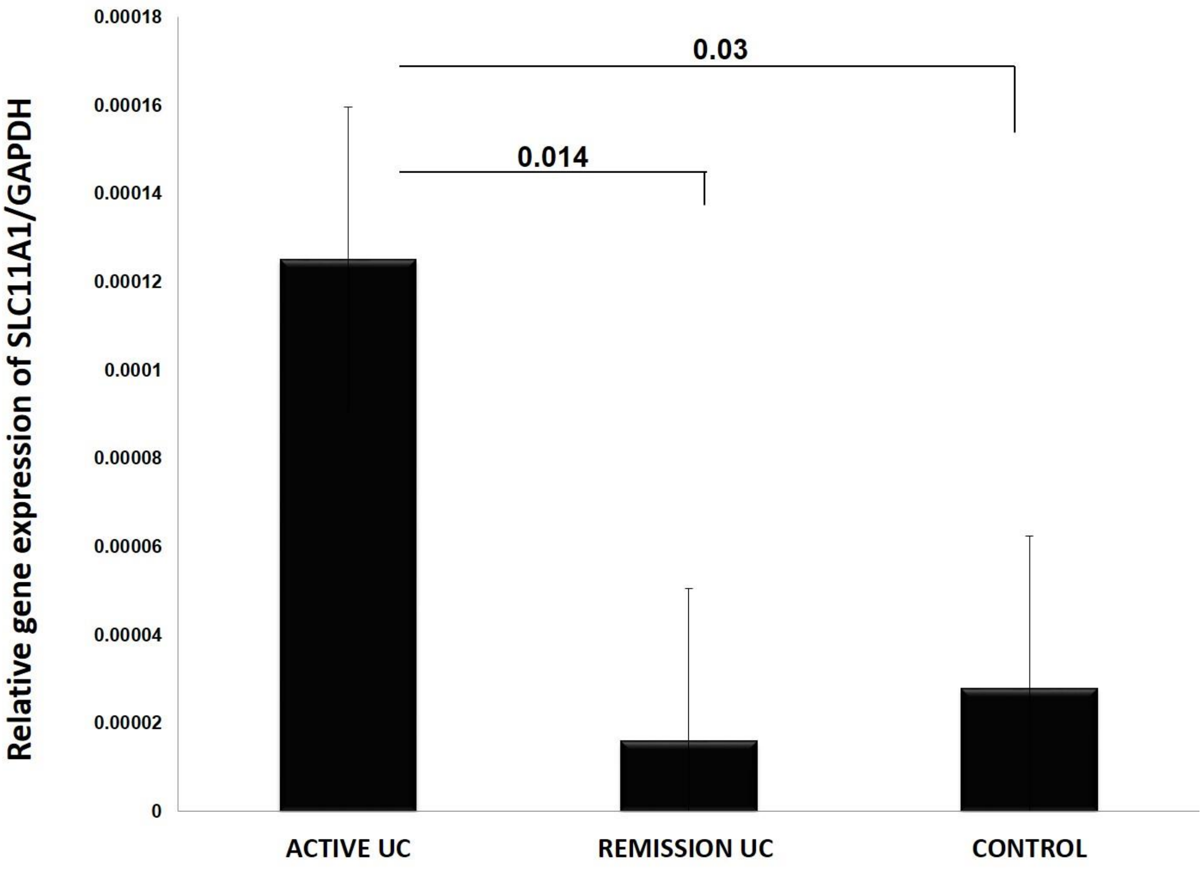

Figure 3s Gene expression of SLC11A1 in colonic mucosa. Results were normalized using GADPH as housekeeping. transcript levels with GAPDH as housekeeping gene determined by 2 Δ ΔCt, differences among groups were assessed by Kruskall Wallis test, and p values are presented in the figure.
